# Supplementary material for: Direct observation of ultrafast singlet exciton fission in three dimensions
Source: Nat Commun. 2022 Oct 10;13:5963. doi: 10.1038/s41467-022-33647-5 (PMC9551063; doi:10.1038/s41467-022-33647-5)
Supplement: Supplementary file 1 — Supplementary Information [file 41467_2022_33647_MOESM1_ESM.pdf]

Supplementary Information for "Direct Observation of Ultrafast Singlet Exciton Fission in Three-Dimensions"

Arjun Ashoka et al.

Supplementary Note 1

Pulse Temporal and Spatial Characterisation

We utilised a homebuilt SHG-FROG to characterise the temporal width of the compressed pump and probe pulses in the sample plane of the objective. Care is taken to ensure that the pulses experience the exact same optical elements and optical path before the SHG-FROG as in the experiment. The retrieved spectra and phases showing transform limited pulses are shown in Supplementary Fig. 1a. The same setup as in [1] was employed. Here the pump spot size at the sample was determined by scanning a 100 nm fluorescent bead across the excitation spot and collecting the flouorescence intensity as a function of bead position (Supplementary Fig. 1b). A Gaussian fit reveals a standard deviation of the pump, sigma, of 115 nm after deconvolution for the 100 nm bead. The overall resolution limit of the setup is given by a convolution between the pump and the diffraction limit of the probe (λ = 750 nm, σ_{pr} = 152 nm of σ_{res} = 190 nm. Refractive index mismatches between the sample and the substrate further increase this limit depending on the magnitude of the mismatch. Imaging aberrations due to linear polarisation of the beams further increase this limit [2].

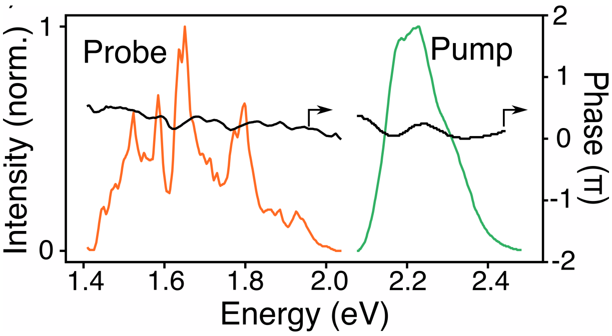

(a) Temporal Characterisation

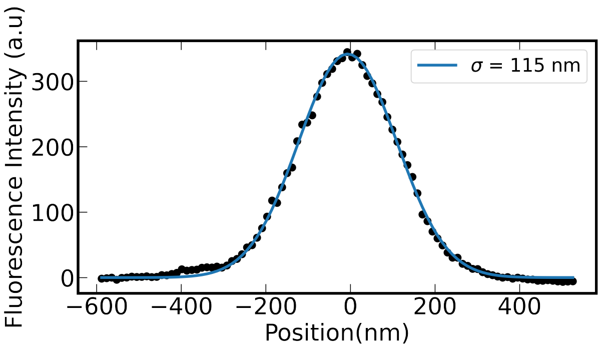

(b) Spatial Characterisation

Supplementary Fig. 1: a) SHG-Frog retrieved spectra of the pump and probe pulses along with their spectral phases, indicating near-transform limited ultrafast pulses (8 fs probe, 13 fs pump). b) Integrated fluorescence intensity profile of a fluorescent bead (TectraSpeckTM Microspheres, T7179) measured by scanning the pump beam across the bead to retrieve the convoluted point spread function.

Supplementary Note 2

Finite Difference Time Domain Benchmark

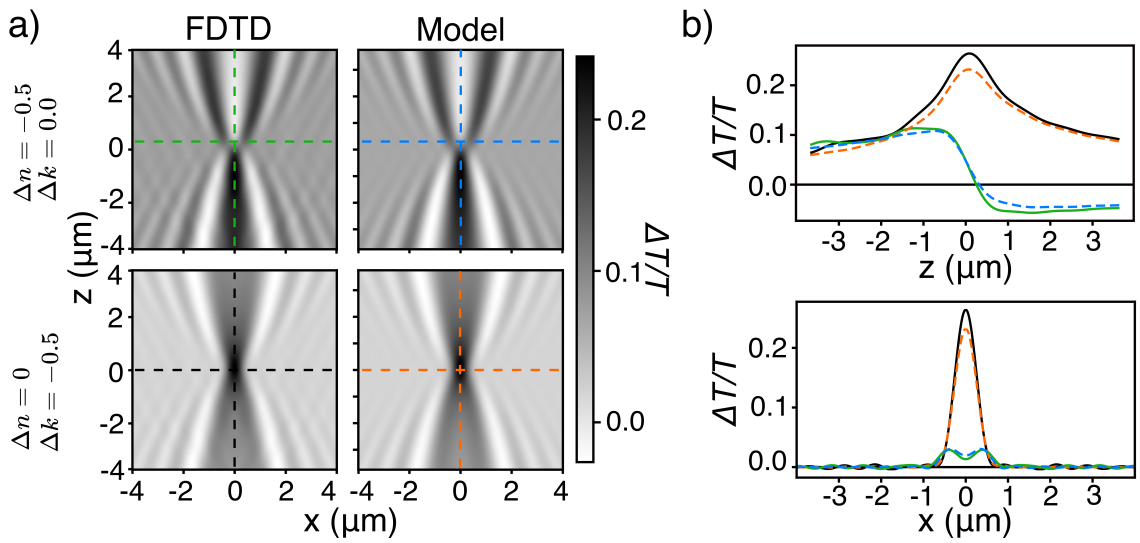

Supplementary Fig. 2: Approximations and benchmark against finite difference time domain (FDTD) calculations. a) Comparison of the optical model and FDTD calculated three-dimensional point-spread of the differential transmission in the far field of a pure refractive and pure absorptive profile. b) Cross-sections along the dotted lines showing excellent quantitative agreement between the optical model and FDTD calculations.

We performed finite difference time domain (FDTD) calculations at a variety of $\Delta\tilde{n}(r)$, sample thicknesses, substrate refractive indices and wavelengths in order to benchmark the validity of our approximations (in particular, Eqn. 5 of the main text) in the true experimental configuration. We have used a disc approximation to the Gaussian and a spatial n,k material, reducing the problem to 2D using cylindrical symmetry. We have used maximal meshing and over 12 perfectly matched layers at the boundary. We ensure the collection of all scattered waves by placing the near field monitor within 1 nm of the sample plane inside the cover-slip glass, ensuring an effective NA of 1.4 and that the refractive index mismatches corresponded to our optical setup. Further, the entire simulation box was over 20 μm wide to ensure that we oversampled the scattered k-vectors. For all simulations here the monitor was placed in a brightfield configuration of a Total-Field-Scattered-Field (TFSF) plane wave source just like our experimental setup. The post processing of the near field was done as per standard Fourier optics, using the Richard-Wolf term for the defocussing [3]. In all cases we find that the nearfield amplitude and phase are very well approximated by Gaussians. In order to highlight any shortcomings of our model we demonstrate here a purely refractive ($\Delta n(r) = -0.5$, $\Delta k(r) = 0$) and purely absorptive ($\Delta n(r) = 0$, $\Delta k(r) = -0.5$) photoexcitation perturbation on a sample of refractive index $\tilde{n} = 1.5+1i$ (Supplementary Fig. 2). We find excellent agreement in the far-field differential transmission images, demonstrating that the near-field approximations are systematically cancelled in the differential far-field images (Supplementary Fig. 2a,b). These results confirm that our simple model manages to capture the relevant optical effects of the local refractive index perturbation and can be used to retrieve the transient optical constants, lateral expansions and vertical shifts in the principal plane from experimental data.

Supplementary Note 3

Uniqueness and comparison to the Gaussian model

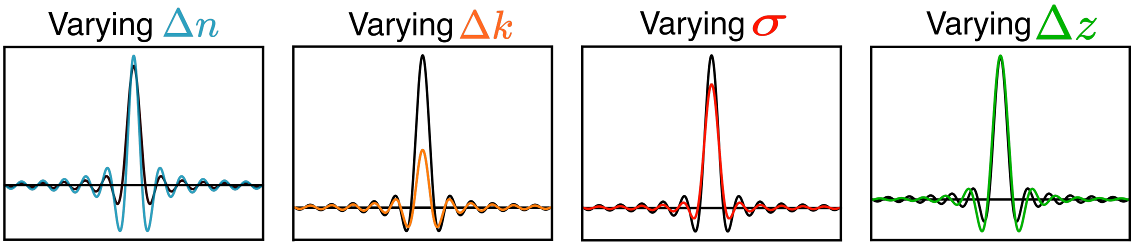

Supplementary Fig. 3: Effect and independence of the parameters of the optical model.

As can be seen in Supplementary Fig. 3, the effect of the 4 time-varying parameters are significantly unique so as to not cause over-fitting within our optical model. This also exemplifies the problems with the Gaussian approximation as it is clear the lateral size of image is strongly influenced by parameters other than σ which is especially clear in the case where Δn is changed. For example, if Δn decreases as a function of time, this will manifest as a spatial expansion through σ in a Gaussian model.

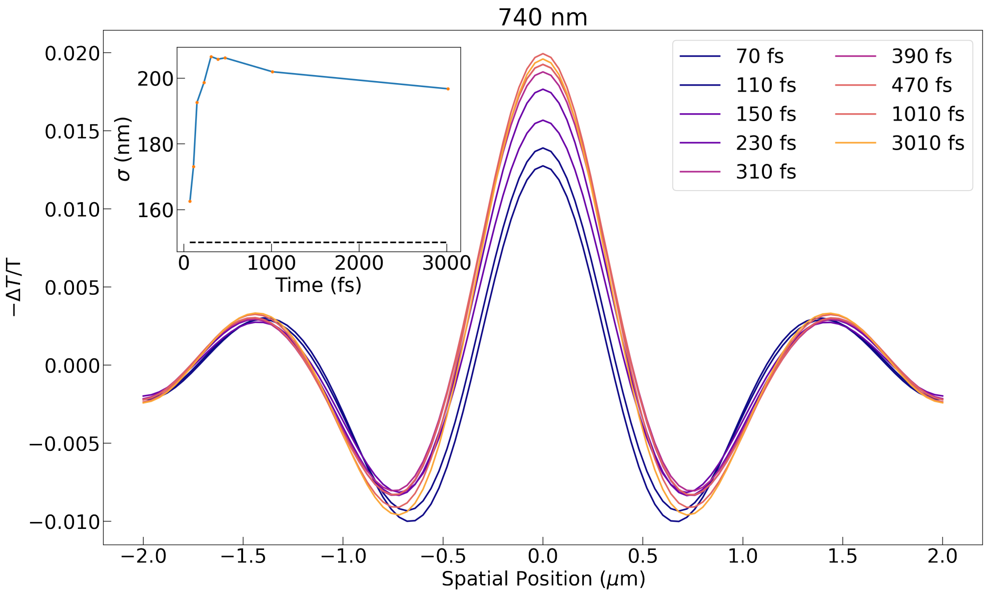

Supplementary Fig. 4: FDTD simulation of the effect of the full complex refractive index on the differential transmission image. Here the near field $n + ik$ is of a constant lateral size σ as a function of time. Inset: Fits to a Gaussian model incorrectly retrieve an expansion in σ of over 40 nm.

In order to bring out this point, we have performed full FDTD simulations of our experiment using the Kramer-Kronig extracted $\Delta n + i\Delta k$ in Pentacene for a fixed Gaussian excitation spot size of $\sigma = 150$ nm at 740 nm. As can be seen in Supplementary Fig. 4, the time dependent images show strong interferometric fringes which are clearly deviating from the Gaussian approximation. From the inset of Supplementary Fig. 4, it is clear that fitting to a Gaussian model retrieves an artificial and incorrect expansion in the lateral σ, which is kept constant in the near field as a function of time. This exemplifies the usefulness of out first-principles facile optical model over the phenomenological Gaussian fit in accurately measuring transport properties.

Supplementary Note 4

Demonstration of Time Resolution

Despite the care taken in the SHG-FROG setup to ensure that identical optical paths when measuring the FROG traces, we additionally show the evolution of the ground-state-bleach signal at 670 nm in Supplementary Fig.5. We chose to use this spectral feature as the rise of the bleach of the ground state should be instantaneous based on the known dynamics of pentacene. Here it can be seen that the signal rises to a maximum over less than 60 fs which yields an instrument resolution of around 15 fs (the stated temporal resolution) as the signal usually takes about $4\tau_{rise}$ to reach 99% of the maximum value.

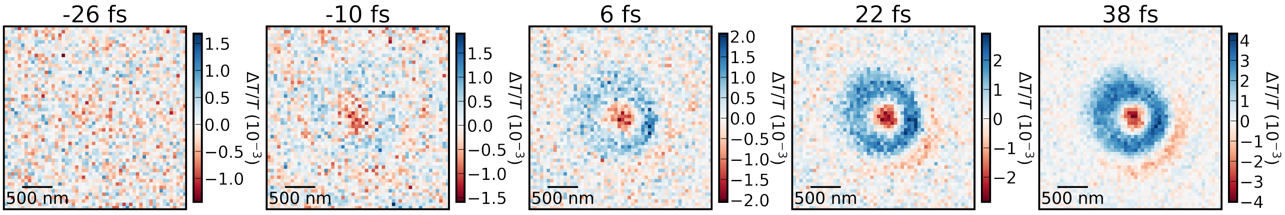

Supplementary Fig. 5: Evolution of the pump-probe image signal over the first 60 fs demonstrating the temporal resolution of the setup in addition to the SHG-FROG characterisation.

Supplementary Note 5

Fitting Procedure

In order to fit the measured images to the interferometic point spread function in Eqn. 7 of the main text and extract $\sigma_{a,c}(t), \sigma_{b,c}(t), \Delta z_b$ and Δz_a we perform the following steps:

1. We define the center of the measured image as the central maximum or minimum signal and then perform a radial average to both improve the signal to noise and to facilitate fitting Eqn. 7 by averaging over the linear polarizations of the pump and probe electric fields.
2. We initialise the global fitting parameters of the sample's static complex refractive index $\tilde{n}_0 = 1.2 + 0.4i$ [4], the substrate refractive index $n_s = 1.5$, the sample thickness $L = 100$ nm, the objectives numerical aperture correcting for the substrate refractive index $NA = 0.9$, a simulation range of -10 to 10 μm and a uniform grid of 5×10^3 points on which the Fourier optics is performed.
3. Using scipy's least squares recursive fitting algorithm and numerical fast Fourier transform (FFT) to compute the predicted radial $\Delta T/T$ image for a given set of time dependent parameters $\Delta n, \Delta k, \sigma, \Delta z$. The resulting image is then interpolated onto the experimental radial grid and then the residuals computed. The residuals are minimised and the error of the fit (reported precision) is calculated by estimating the covariance matrix using the Jacobian output by scipy.
4. The final fit parameters at a given time point are taken as the guess for the next time point and the previous step is repeated until the final time point of the measurement is reached, yielding $\Delta n(t), \Delta k(t), \sigma(t), \Delta z(t)$.

To extract $\sigma_{a,c}$ and Δz_b, the a,c plane of pentacene should be in the plane of the microscope objective and the b-axis out of plane. This is the scenario on the glass substrate as shown in Fig. 1 of the main text. In order to extract $\sigma_{b,c}$ and Δz_a, the b,c plane of pentacene should be in the plane of the microscope objective and the a-axis out of plane. This is the scenario on when pentacene is grown in hBN [5]. Hence both measurements must be performed and can be used as benchmarks for each other if the underlying measured transport is linked to the crystal direction. This is the procedure underlying the data shown in each of the Fig. 2-4 of the main text. We note that due to the singlet and triplet electronic polarisation dipoles are orthogonally oriented in the pentacene crystal axis and therefore based on the wavelength being measured a half-wave plate in the beams paths was adjusted to maximise the pump-probe signal.

Supplementary Note 6

Axial and Lateral Localisation Precision

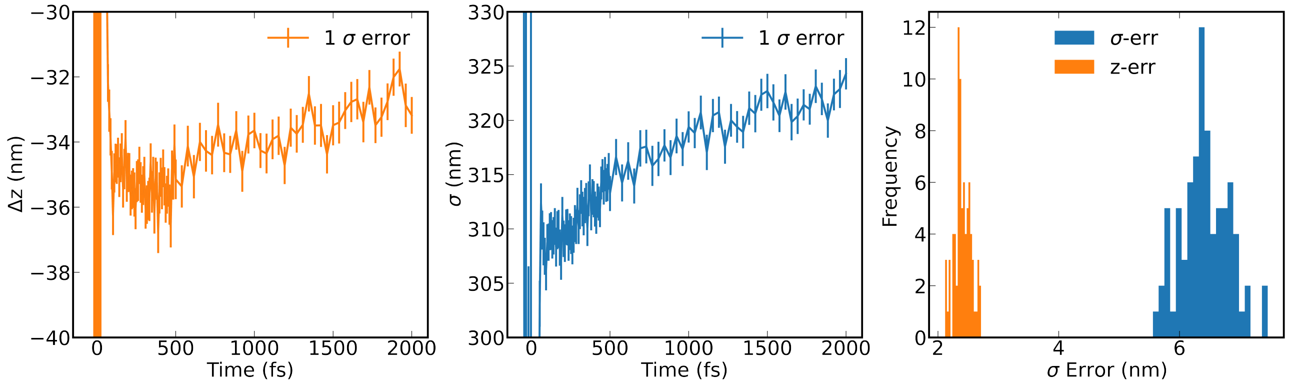

Supplementary Fig. 6: The extracted $\Delta z(t)$ and $\sigma(t)$ from the measurement along with their 1 σ values from the Hessian of the Jacobian of the fit and histograms of the errors for the high fluence measurement at 740 nm (Fig. 4 of the main text).

Calculating the localisation precision in single molecular microscopy is done by measuring the scatter of localisations when a molecule is imaged and localised repeatedly. This is fundamentally limited by the signal to noise ratio (SNR) and not by the wavelength of light or the pixel size [6]. In our particular experiment of interferometic localisation microscopy of a fixed excitation as a function of time-delay, the localisation precision at a given pump-probe delay can be analogously estimated by the standard deviation of the result many measurements. Alternatively as our experiment is a time series measurement, the peak-to-peak, high frequency noise on the time series can itself provide an estimator for the localisation precision as has been reported in the past [7].

A third critical consideration is that the localisation extracted in our experiments are done through the use of model fits to experimental data. Hence the 1σ confidence interval of the final fit parameters are likely the most critical estimators of the true localisation precision. The difficulty arises in defining these estimators correctly, as fits of Gaussians to interferometic point-spread-functions in the past have claimed very high lateral precision based on this while the model clearly does not capture all the features of the data and has, for example, a low R^2 coefficient [8, 9]. This is akin to using single straight line to describe a complex curve and using the stability of the fit to claim confidence and precision in the model's fit.

Hence we stress that our first-principles analytic optical model is far superior at estimating the precision accurately as all the features of the data are captured well including the interferometic contrast fringes. As we are solving a minimisation problem,

$$\min_x \frac{1}{2}||y - f(x)||^2,$$

we take a Taylor series of f:

$$f(x) = f(x_0) + J(x - x_0) + \mathcal{O}(x^3).$$

The approximate optimisation problem formed by truncating the Taylor series,

$$\min_x \frac{1}{2}||y - f(x_0) - J(x - x_0)||^2,$$

has Hessian $J^T J$ when the solution is fully converged.Using the Jacobian given by Scipy's least squares optimisation algorithm we therefore estimate the covariance matrix as $J^T J$ and report the 1σ intervals in Supplementary Fig. 6. As can be seen, in the best case scenario our localisation precision bound from the confidence interval of the fit is less than 5 nm in the axial direction and less than 10 nm in the lateral direction. This is in line with other state-of-the-art static interferometric scattering models [10].

Supplementary Note 7

Remote focusing for z-sectioning

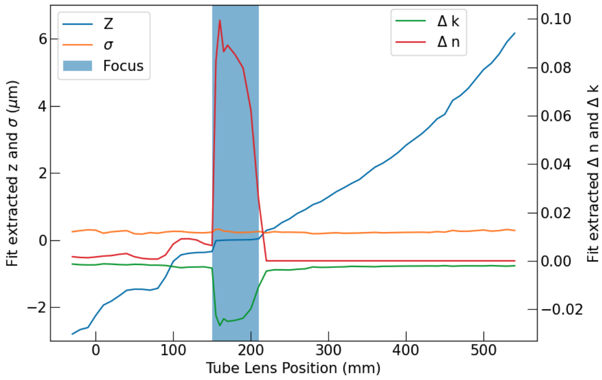

(a) Remote Focussing Parameters

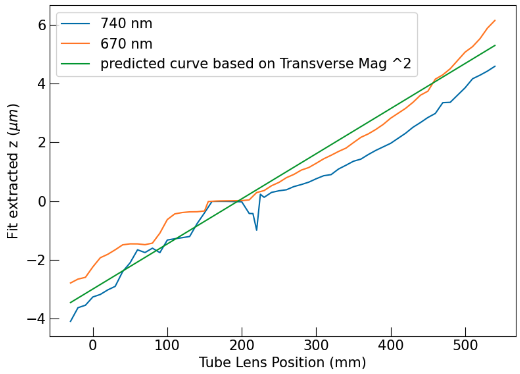

(b) Remote Focussing Axial Magnitification

Supplementary Fig. 7: a) Fits of the images in the z-stacks displayed in Fig 1c of the main text at 670 nm displays that the near field σ is insensitive to the imaged plane (orange line) and that the retrieved Δn and Δk are correct only within the axial focal region. b) Characterisation of the axial magnification and chromatic aberrations of the system using fits to the z-stacks reported in Fig 1c of the main text.

We have fitted the images in the z-stacks displayed in Fig. 1c of the main text to our developed optical model in order to bring out the limitations of our technique using the remote focusing method. Here we have used unbounded fits with a fixed initial guess with the correct signs for Δn and Δk to test the efficacy of the developed optical model. As can be seen in Supplementary Fig. 7a, using our model, the near field σ is critically insensitive to the imaged plane (orange line). Further, we find that the effect of a defocus in the object plane can be adequately captured by moving the imaging lens to focus different image planes on the camera. This is exemplified by the linear relationship between the fit parameter z and the tube lens position shown in Fig 7a. It is however, critical to understand the accuracy of our model in retrieving the transient complex refractive index as a function of axial defocus in order to pick a suitable interferometric imaging plane. As shown in Fig 7a, only within the axial focus of the objective is the model well behaved. It is also important to note that, as expected, the lateral size of the near field Gaussian that is retrieved by the model is insensitive to the selected imaging plane.

In order to characterise the relation between the tube lens position and the imaged plane we repeat the same measurement at two imaging wavelengths (Fig 7b). Here we can see that the predicted axial magnification = (transverse magnification)^2 is obeyed as the transverse magnification has been characterised as 255 for our system. Further, the $\mathcal{O}(\mu m)$ offset between the retrieved imaging plane positions for the different wavelengths is a sign of the significant chromatic aberrations in our system which may actually aid in interferometric contrast [11].

Supplementary Note 8

Atomic Force Microscopy of Microcrystalline Pentacene Films on Glass and hBN

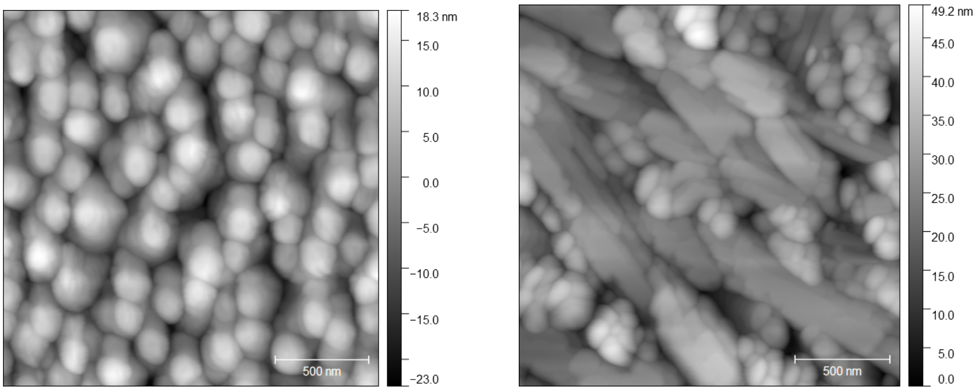

(a) Pentacene on Glass

(b) Pentacene on hBN

Supplementary Fig. 8: AFM images of microcrystalline pentacene on a) Glass and b) hBN, demonstrating orthogonal domain crystallisation with domain sizes larger than 200 nm.

AFM is measured in contact mode (Asylum Research MFP-3D). As can be seen in Fig 8, the microcrstalline domains are orthogonal with respect to the substrate when evaporated on glass or hBN, in line with previous reports [5]. The domain sizes are larger than 200 nm which allows us to photoexcite a single domain using our 100x 1.1NA microscope objective.

Supplementary References

[1] Sung, J. *et al.* Long-range ballistic propagation of carriers in methylammonium lead iodide perovskite thin films. *Nature Physics* **16**, 171–176 (2019).

[2] Diel, E. E., Lichtman, J. W. & Richardson, D. S. Tutorial: avoiding and correcting sample-induced spherical aberration artifacts in 3d fluorescence microscopy. *Nature Protocols* **15**, 2773–2784 (2020).

[3] Electromagnetic diffraction in optical systems, II. structure of the image field in an aplanatic system. *Proceedings of the Royal Society of London. Series A. Mathematical and Physical Sciences* **253**, 358–379 (1959).

[4] Hinderhofer, A. *et al.* Optical properties of pentacene and perfluoropentacene thin films. *The Journal of Chemical Physics* **127**, 194705 (2007). https://doi.org/10.1063/1.2786992.

[5] Amsterdam, S. H. *et al.* Tailoring the optical response of pentacene thin films via templated growth on hexagonal boron nitride. *The Journal of Physical Chemistry Letters* **12**, 26–31 (2020).

[6] Lelek, M. *et al.* Single-molecule localization microscopy. *Nature Reviews Methods Primers* **1** (2021).

[7] Pandya, R. *et al.* Microcavity-like exciton-polaritons can be the primary photoexcitation in bare organic semiconductors. *Nature Communications* **12** (2021).

[8] Schnedermann, C. *et al.* Ultrafast tracking of exciton and charge carrier transport in optoelectronic materials on the nanometer scale. *The Journal of Physical Chemistry Letters* **10**, 6727–6733 (2019).

[9] Delor, M., Weaver, H. L., Yu, Q. & Ginsberg, N. S. Imaging material functionality through three-dimensional nanoscale tracking of energy flow. *Nature Materials* **19**, 56–62 (2019).

[10] Mahmoodabadi, R. G. *et al.* Point spread function in interferometric scattering microscopy (iSCAT) part i: aberrations in defocusing and axial localization. *Optics Express* **28**, 25969 (2020).

[11] Sheik-Bahae, M., Said, A., Wei, T.-H., Hagan, D. & Stryland, E. V. Sensitive measurement of optical nonlinearities using a single beam. *IEEE Journal of Quantum Electronics* **26**, 760–769 (1990).
